# A pilot study investigating the acceptability and feasibility of a group intervention for parents of autistic young people with anorexia nervosa within a specialist eating disorders service

**DOI:** 10.1186/s40337-025-01399-4

**Published:** 2025-09-29

**Authors:** Sofia Loizou, Kate Beresford, Julian Baudinet, Paula Herrera-Gener, Danielle Oregan, Colleen Alford, Kate Tchanturia, Catherine Stewart

**Affiliations:** 1 King’s College London, London, UK; 2 South London and Maudsley NHS Foundation Trust, London, UK; 3 Eating Disorders Service, Sydney Children’s Hospitals Network, Sydney, Australia

**Keywords:** Anorexia nervosa, Autism, Carers, Parents, Intervention

## Abstract

**Background:**

Many children and young people presenting with anorexia nervosa will also be autistic. While autistic children and young people may have similar physical health outcomes compared to their neurotypical peers, they are more likely to experience poorer recovery, requiring more intensive treatments. Similarly, parents report needing additional support from mental health services. Parents may benefit from a group intervention as an adjunct to family therapy to help them effectively care for their children and to reduce the psychological impact this may have on them. This study aimed to provide an overview of the group intervention and evaluate its feasibility and acceptability.

**Methods:**

Attendance rates were used to evaluate the feasibility of the group. Parents were invited to evaluate the acceptability of the group with brief quantitative measures each session. Individual qualitative interviews were conducted with six parents to evaluate both feasibility and acceptability. Recordings were transcribed verbatim and analysed using reflexive thematic analysis.

**Results:**

17 parents (mothers *n* = 8, fathers *n* = 8, stepmother *n* = 1) from eight families took part in two pilot groups (group 1 *n* = 8, group 2* n* = 9). Total attendance rate was 66.5%, with one parent from each family attending 85.7% of sessions. On average, sessions were rated highly relevant, useful and improved parents’ understanding of their child. From qualitative interviews conducted with six parents (group 1 *n* = 4, group 2 *n* = 2), two main themes were generated: (1) A space for connection and support, (2) From knowledge to practice. Parents spoke of the way the group helped them feel less isolated, gain skills and begin to practice implementing new learning at home. Not all aspects of the group were helpful, and recommendations were made regarding the resources, structure and intensity of the intervention.

**Discussion:**

Findings suggest that the intervention is feasible and acceptable. This pilot study replicates previous findings about benefits of additional support for carers.

**Supplementary Information:**

The online version contains supplementary material available at 10.1186/s40337-025-01399-4.

## Plain language summary

The aim of the current study was to test a new group intervention aimed to support parents of autistic young people with anorexia nervosa. Parents rated each group session and were interviewed to gather feedback on their experiences of attending the group intervention. Most parents who took part in the interviews generally described the group as helpful, particularly in providing a sense of connection and identifying practical ways to support their autistic children with their eating. The positive feedback highlights the group’s potential value. Parents described several challenges to attending and fully engaging with the group, including work and caring commitments, and differences in relating to others who might have had different experiences. Suggestions to improve the group included developing and sharing electronic handouts and offering a follow-up session to reinforce the learning. Parents also felt the group would be more helpful if it was offered earlier in their child’s treatment.

**Supplementary Information:**

The online version contains supplementary material available at 10.1186/s40337-025-01399-4.

## Introduction

There is increasing recognition of a high prevalence of autism and autistic traits in children and young people (CYP) with anorexia nervosa (AN) [13, 57]. It has been hypothesised that autism and AN share a genetic vulnerability [13, 67], which may result in overlapping neurological or hormonal differences (e.g., changes in oxytocin or interoception) [1]. In the adult clinical population approximately 10% of people with AN who are attending eating disorder services may meet the threshold for an autism diagnosis, while around 40% present with autistic traits [13, 64, 68].

Available data suggests there is an overlap between AN symptoms and autistic traits. These involve difficulties in several domains including emotion recognition [16, 21, 33, 34, 42], theory of mind [19, 59], emotional introspection [34], social skills [4, 34], attention-switching and flexibility [34, 40, 69, 70], perspective-taking [34], tolerance of uncertainty [29] and sensory sensitivities [38]. It has been proposed that starvation may influence some of these traits [65], however, these appear to persist following weight restoration for some and have been observed before the onset of starvation [22, 23, 66].

Eating disorder focused family therapy is the first-line recommended treatment for child and adolescent eating disorders across most international guidelines [20, 30, 31, 50]. There are several manualised versions of this treatment, including Family Therapy for anorexia nervosa (FT-AN) [25], Family-Based Treatment (FBT [46]) and Parent-Focused Therapy (PFT [41]). Across these, the shared aim of treatment is to support the young person with the support of the family system. The FT-AN manual outlines a typical treatment duration of between six and nine months [25]. Sessions begin on an intensive weekly basis but transition to fortnightly before extending to monthly (or beyond) as progress is made.

Evidence from randomised controlled trials (RCTs) demonstrates that family therapy is an effective intervention for the treatment of AN (cf., [5] for review). However, many will not recover with family therapy alone [45] and may require more or different support. Whilst data suggests autistic CYP experience similar physical health outcomes in AN recovery to their neurotypical peers, they may report sustained eating disorder cognitions [62]. In a large Swedish study of long-term outcomes for teenage-onset AN, those with autism showed poorer Morgan-Russell outcomes [49], specifically across mental, psychosexual and socio-economic states [53]. Additionally, autistic adults with AN reported that standard treatments do not always meet their needs, acknowledging that difficulties with rigidity, flexibility and social processing can contribute and maintain AN [36]. Both autistic adults with AN and carers of autistic adolescents and young adults (16–25 years) with AN have highlighted that adapting treatments may help to improve outcomes [2, 6, 36]. Individualised, collaborative formulation may help [11], however, there are currently no evidence-based guidelines on how to best to adapt AN treatment for autistic people [50]. Clinicians also describe hesitance to make adaptations to treatment as they fear potential harm [24].

Given parents play a key role in eating disorder focused family therapy, more intensive support offered for parents of autistic CYP may be one way to increase support and improve outcomes. Qualitative research has highlighted the lack of support for parents/carers of autistic adolescents and adults (12–36 years) with AN [2, 37, 54]. Parents described feeling frustrated and isolated, and unable to work because of increasing caring demands [2]. Carers have described difficulties linked to having additional needs that may not be addressed by eating disorder treatments, feeling unsupported by services, and have expressed that caring for their children can have a significant emotional and financial impact on them [37]. The lack of existing support systems has also prompted carers to set up their own support groups and networks online [2].

Group interventions may be helpful in providing parents with increased support and a reduced sense of isolation through the opportunity to learn new skills and process difficulties with other parents in similar situations. Within the adult sector, the Maudsley eating disorder collaborative care skills workshops aimed to improve carers’ sense of competency and expressed emotion, whilst reducing caregiver distress, burden and specific difficulties associated with eating disorder symptoms [60, 61]. Greater parental intolerance of uncertainty is thought to decrease parents’ confidence to care for their children with AN [39] and may be ameliorated in a group-based context. High levels of parental self-efficacy and lower levels of expressed emotion have also been associated with improved treatment response and outcomes in family therapy for eating disorders (e.g., greater weight gain) [12, 18, 26, 58]. These findings suggest that group interventions for parents/carers may equip them with skills and support them to effectively care for their children and consequently facilitate recovery and maximise outcomes.

Loomes and Bryant-Waugh [47] have suggested that parents of autistic CYP may benefit from additional psychoeducation on AN and autism, emotion coaching, and distress tolerance skills. Additionally, they suggest making accommodations to support autistic CYP’s traits such as intolerance of uncertainty, difficulties with flexibility and preference of routine.

Considering the literature and these recommendations, a brief parent group was developed for parents of autistic young people with AN. This group may be particularly useful for families transitioning to later phases of FT-AN (around phase 2–3). Phase 2 of FT-AN involves supporting the family to effectively manage the eating disorder symptoms (including weight restoration, physical recovery, habituation towards flexible eating patterns through reactivating positive parental and relational patterns). Phase 3 of FT-AN involves exploring issues of individual and family development (including addressing intolerance of uncertainty, handing back responsibility, reviewing changes in family relationships and the family life cycle). At this point in treatment, families should be attending sessions less frequently. Consequently, parents could transition to broader discussions around individual and family identity in the group context.

This pilot study aims to:

1. Provide an overview of a group intervention for parents of young people with AN and suspected or diagnosed autism at the Maudsley Centre for Child and Adolescent Eating Disorders (MCCAED).
2. Evaluate the feasibility and acceptability of the parent group intervention.

## Methods

This project was approved by the South London and Maudsley NHS Foundation Trust Service Evaluation and Audit Committee (ID 421).

### Design

Data were collected for the two pilot groups offered at MCCAED. The first group ran from November to December 2023 and the second from March to April 2024. Session-by-session rating scales were used to assess acceptability. Individual qualitative interviews were offered at the end of the intervention to gather an in-depth understanding of parent experiences.

Quantitative data (pre/post measures) have also been collected. Quantitative findings will be published once further data are collected as the sample size is too low for meaningful analysis.

### Sample

Parents of young people with AN with a diagnosis of autism or suspected autism who were in late Phase 2 or Phase 3 of FT-AN were eligible for the group. The decision to include parents of CYP with suspected autism, rather than solely those with a confirmed diagnosis, stemmed from the long waitlists to receive an autism diagnosis in the NHS (NHS [51]).

### Procedure

MCCAED clinical staff identified parents of young people with AN and diagnosed or suspected autism receiving FT-AN who could benefit from the intervention. An email containing relevant information was shared with parents expressing interest in joining the group. Parents were asked to complete a range of instruments, including self-report questionnaires pre- and post-intervention, session-by-session rating scales (online via the Qualtrics platform or using paper and pen forms). All parents who attended the group intervention were also invited to take part in individual interviews to share their experiences of the group and ideas on how the group could be improved. Those who expressed an interest met with a Trainee Clinical Psychologist via Teams or Zoom to conduct the interview, lasting approximately 30–60 min.

### Intervention

The group intervention consisted of seven two-hour weekly sessions. They were facilitated by clinical psychologists (all sessions), a psychiatrist (all sessions), two parents with lived experience (one session) and a dietitian (two sessions). A session-by-session outline is provided in Table 1. Topics covered in the group included:

- Psychoeducation on autism and AN was included to build a deeper understanding of the unique challenges facing this cohort (particularly for girls). This included diagnostic overshadowing, delayed diagnosis and the impact of masking. Information about differences in sensory processing, flexibility, interoception and social interaction and communication was also incorporated to empower parents to appreciate strengths and understand stuck points impacting treatment.
- Information about nutrition was provided and practical strategies were discussed to support parents with mealtimes. These strategies aimed to help parents make accommodations for autism, whilst also addressing rigidity related to AN.
- Distress tolerance and emotion regulation skills were taught to help parents recognise distress perpetuated by AN and unmet needs related to autism and respond effectively.
- Information about parental self-care was included to allow parents to reflect on their own unique needs (including navigating the healthcare system, gaining access to appropriate services, and feeling isolated or misunderstood by others). Parents were supported with specific knowledge and skills to manage their own wellbeing, enhancing their ability to function at home and at work.
- Parents with lived experience co-facilitated a session to offer a space for parents to connect and reduce feelings of isolation, blame and guilt. The aim of having a lived experience family was also to instil hope and to help parents identify their child’s needs. Through lived experience input it was also hoped that parents would develop recovery expectations that were neuro-affirming and not perpetuating neuro-normative ideals about food and eating.
- Discussion of endings was included to help parents acknowledge and process anxiety shared between the young people and their families about an impending transition; both from the parent group and treatment for AN broadly.
- Resources were provided to support knowledge and understanding. Some were tailor-made for the intervention. Others were taken (with permission) from the PEACE (Pathway for Eating disorders and Autism developed from Clinical Experience) pathway (https://peacepathway.org/patients/resources).
- Homework was given between sessions to reinforce learning and integrate this to their own environment, as well as build confidence in parents’ ability to implement new strategies and support long-term change. Homework was closely related to the content covered in the sessions.
- Opportunities to interact with other parents and share their experiences were provided at times to increase a sense of connection and support.

**Table 1:** Autism parent group content

Sessions	Group 1 content	Group 2 content	Example activity
1. Autism and anorexia	Introduction to autism, autistic females, anorexia and dual diagnosis, shared neurobiology	Introduction to autism, autistic females, anorexia and dual diagnosis, lived experience parent session	*Reflections on autism—*Families reflect upon their young person’s traits, strengths and differences
2. Understanding emotions and managing distress	Shared neurobiology, function of AN with autism, understanding emotions, strategies for managing distress	Shared neurobiology, function of AN with autism, understanding emotions, strategies for managing distress	*Emotion scale—*Tool to build collaborative understanding of emotional needs and supports to regulate emotions
3. Managing meals	With Dietitian—Recovery from anorexia when autistic—strengths and challenges, window of tolerance, sensory sensitivities, interoception	Recovery from anorexia when autistic—strengths and challenges, window of tolerance, sensory sensitivities, interoception	*Sensory summary—*PEACE pathway tool to determine sensory needs for the young person. Parents to reflect on shared and competing sensory needs in the family
4. Managing meals	Dietitian-led—Understanding baseline nutrition, realistic nutrition goals, how to challenge new foods/environments	Dietitian-led—Understanding baseline nutrition, realistic nutrition goals, how to challenge new foods/environments	*Setting a realistic baseline—*Reflecting on diet (volume, variety, flexibility, sensory needs, social eating) prior to development of AN. How are needs similar and different?
5. Adolescence	Understanding adolescence and attachment needs, relational sequence, self and identity, peer relationships	Understanding adolescence and attachment needs, relational sequence, self and identity, peer relationships	*Iceberg sequence—*Complete a relational sequence focused on a recent episode of distress, being mindful of new knowledge around AN and autistic traits
6. Broader needs	Stressors and strains, carer styles, self-care	Unique parenting needs, broader family support, self-care	*Family and social circles—*Explore social networks of support in autistic families—have these been shaped by AN? Increase parental self-care
7. Recovery and endings	Remaining needs, lived experience parent session, tolerating transitions and endings	Remaining needs, tolerating transitions and endings	*Communication passport—*PEACE pathway (https://www.peacepathway.org) tool for young person to communicate needs (sensory, relational, communication) with ongoing supports [43]

Several modifications were made to the content and structure of the group based on feedback from the first group to improve flow and engagement (see Table 1). For instance, the lived experience parent session was moved forward to introduce a sense of hope early in the group about the possibility of change. The first cohort reflected that this session raised affect, inspired new thinking and prompted questions which suggested families would benefit from attending this session earlier on in the group.

### Measures

#### Feasibility

Attendance rates were used to evaluate the feasibility of the group.

#### Acceptability

Parents were asked to rate three questions on a 10-point likert scale after each session, to evaluate the acceptability of the group:

- Was it relevant? (0 = not at all, 10 = very)
- Was it useful? (0 = not at all, 10 = very)
- Did it improve your understanding of your child? (0 = not at all, 10 = significantly)

Parents from both groups had the opportunity to partake in qualitative semi-structured interviews exploring their views and experiences of the group. An interview schedule was developed by the team to guide interviews with parents (interview schedule can be found in supplementary material).

### Lived experience involvement

Two parents from the same family with lived experience of caring for an autistic child with a diagnosis of AN reviewed the study aims/objectives and co-wrote the content of the intervention. Their feedback and insights were invaluable in ensuring that the content would align with the needs of families. They also co-delivered a session, where they shared their experience and participated in discussion with group members. The two parents also provided feedback on the interview schedule to ensure that it covered all important areas/topics and questions were phrased in a sensitive manner.

### Analysis and reflexivity statement

Qualitative interviews were transcribed verbatim and analysed using reflexive thematic analysis [14] informed by a critical realist approach [28]. This involved the following steps: (1) familiarisation with the data, (2) generating codes; (3) developing initial themes; (4) reviewing themes; (5) refining themes and (6) write up [14]. Transcripts were coded in Microsoft Word and then transferred into Excel to organise and sort themes.

SL (female, White, Cypriot, Trainee Clinical Psychologist) conducted and transcribed interviews. Coding and theme generation were conducted by authors SL and KB (female, White, Australian, Clinical Psychologist). While a predominantly inductive approach was taken to develop the themes, the authors’ experiences and background have influenced the study findings and their interpretability. At the time of analysis, SL was a Trainee Clinical Psychologist on placement in MCCAED. KB had several years of clinical experience delivering FT-AN and working with young people with eating disorders in the UK and Australia. She was a facilitator in both groups. SL attended two sessions from the second group to familiarise herself with the content, format and process. The authors acknowledge that the involvement of coders in the delivery of the intervention may have influenced the generated themes, potentially introducing a positive bias. The authors also recognise that participants may have felt hesitant to express negative views, given that families could have still been under the care of MCCAED.

## Results

### Participants

Seventeen parents (mothers *n* = 8, fathers *n* = 8, stepmother *n* = 1), of eight CYP took part in the intervention. Eight parents (from four families) attended group 1 and nine parents (from four families) attended group 2. CYP had a mean age of 14.50 (*SD* = 1.51). Six (75%) CYP were White British and five (62.50%) had an autism diagnosis. More information can be seen in Table 2.

**Table 2:** Participant characteristics

Characteristics	Group 1 *N* (%) (*n* = 8)	Group 2 *N* (%) (*n* = 9)
Age range—child	12–15 years (*M* = 14.25, *SD* = 1.50) (n = 4)	13–17 years (*M* = 14.75, *SD* = 1.71) (n = 4)
*Gender—parent*		
Female	4 (50%)	5 (55.55%)
Male	4 (50%)	4 (44.44%)
*Gender—child*		
Female	3 (75%)	4 (100%)
Male	1 (25%)	–
*Ethnicity—child*		
White British	3 (75%)	3 (75%)
White other	–	1 (25%)
Mixed	1 (25%)	–
*FT-AN phase*		
Phase 2	2 (50%)	2 (50%)
Phase 3	2 (50%)	2 (50%)
*Autism diagnosis*		
Diagnosis	3 (75%)	2 (50%)
Suspected	1 (25%)	2 (50%)

### Feasibility

Accounting for both groups, the total attendance rates was 66.39%. Overall, one parent from each family attended 85.71% of sessions. Further information can be seen in Table 3.

**Table 3:** Attendance rates

	Session 1	Session 2	Session 3	Session 4	Session 5	Session 6	Session 7	Total
Group 1 (*n* = 8)	5 (62.50%)	6 (75%)	5 (62.50%)	6 (75%)	5 (62.50%)	7 (87.50%)	4 (50%)	(67.86%)
At least one parent per family (Y = yes, N = no)	Y	Y	Y	Y	Y	Y	Y	100.00%
Group 2 (*n* = 9)	8 (88.88%)	6 (66.66%)	5 (55.55%)	6 (66.66%)	6 (66.66%)	3 (33.33%)	7 (77.77%)	(65.08%)
At least one parent per family (Y = yes, N = no)	Y	Y	N	N	Y	Y	Y	71.43%

In group 1, at least one parent from each family attended all sessions. In group 2, at least one parent from two families attended all sessions, while at least one parent from each family attended six out of seven sessions and at least one parent from each family attended four out of seven sessions.

### Acceptability

On average, sessions were rated highly relevant (*M* = 8.61, *SD* = 1.60), useful (*M* = 8.58, *SD* = 1.63) and improved parents’ understanding of their child (*M* = 7.71, *SD* = 2.18). More details can be found in Table 4.

**Table 4:** Mean ratings for each session

	Was it relevant? *M* (*SD*)	Was it useful? *M* (*SD*)	Did it improve your understanding of your child? *M* (*SD*)
Session 1	8.50 (1.38) (*n* = 6)	8.83 (0.98) (*n* = 6)	7.50 (1.64) (*n* = 6)
Session 2	8.40 (1.52) (*n* = 5)	8.40 (1.14) (*n* = 5)	6.80 (1.79) (*n* = 5)
Session 3	7.80 (2.59) (*n* = 5)	7.20 (2.59) (*n* = 5)	6.60 (3.21) (*n* = 5)
Session 4	8.86 (1.86) (*n* = 7)	8.43 (1.81) (*n* = 7)	7.43 (2.44) (*n* = 7)
Session 5	8.50 (0.71) (*n* = 2)	9 (1.41) (*n* = 2)	9 (1.41) (*n* = 2)
Session 6	8.50 (0.71) (*n* = 2)	9 (1.41) (*n* = 2)	8.50 (0.71) (*n* = 2)
Session 7	9.75 (0.50) (*n* = 4)	10 (0) (*n* = 4)	10 (0) (*n* = 4)

### Qualitative interviews

Six parents (group 1 *n* = 4, group 2 *n* = 2) took part in qualitative interviews. Parents consisted of four mothers (66.67%) and two fathers (33.33%), including two mother–father dyads each corresponding to a distinct family unit and child. Two themes were generated using reflexive thematic analysis [14].

#### Theme 1: A space for connection and support

This theme underscores concepts of connectedness, shared learning and having a safe space to express and process difficult emotional reactions related to AN and/or autism diagnosis, caring demands and navigating the healthcare system. It also emphasises the importance of prioritising parents as experts, and highlights factors influencing engagement and participation, along with potential areas for improvement.

*Subtheme 1.1: Connection and solidarity; but is it enough?*

Parents described a sense of relief and gratitude for having a space to share their experiences with others. For many parents, the group was a great source of support and solidarity, contributing to a decrease in loneliness and isolation. Several parents reported that having a supportive space allowed them to express their frustration about how standard care without adaptations for neurodiversity felt lacking at times for their young person or family needs. Interactions between parents also led to reductions in guilt and self-blame by recognising that autism and AN can present in families of all backgrounds.

*I think it was greatly helpful and very cathartic to hear other people's experiences and to feel supported when you're talking about your own experiences and, you know, I think there's a great deal of, you know, great deal of empathy in the room. (Parent 4, Group 1).*

*If you're in a group and suddenly you were sort of freakishly all the same, you're saying “oh God, it is our fault because we are all like this?” So, it helps you sort of think that it's not, you know, it helps to the extent that you kind of blame issues on yourself (Parent 5, Group 1).*

For families further along in their treatment journey, the intervention did not always fully align with their current needs. Parents expressed greater feelings of isolation and a stronger need for additional support in the earlier stages of FT-AN.

*We felt very isolated at the time, so it was a new kind of reaching out for things and we had a smaller network then, so specifically when it just started, when it just hit us, so it was quite new to us having a daughter with anorexia. It was, you know, it's like can we speak to some other parents just to sort of centre ourselves somehow, you know? (Parent 5, Group 1)*

*I wish this group was like before we even started this process, because that would have been a bit more helpful, I guess. (Parent 3, Group 2)*

Parents also reflected on broader systemic limitations impacting expectations of their child’s treatment network, including high resource needs. Equally, they recognised the way professionals and parents alike were having to continually adapt their thinking and practice alongside emerging guidelines.

*A general experience of the authorities is quite poor. I would say that most of the people [professionals] mean very well, and individually they're all quite nice. Very nice. But it's very disjointed. It's very under-resourced. They don't seem to know - they don't really know how to navigate it themselves. (Parent 5, Group 1)*

Several parents reported barriers to attending the group including work and caring demands. There were mixed views as to whether face-to-face or remote sessions would be more appropriate. On one hand, face-to-face sessions could offer the opportunity to develop deeper connections and more dynamic engagement, but on the other hand, remote sessions could offer greater accessibility and flexibility for those that could not attend in person.

#### It's very difficult to come meet and come every week, I think I missed a session. (Parent 3, Group 2)

*Subtheme 1.2: Experts by experience*

Parents said that having parents with lived experience co-facilitate the group was also important in instilling hope. However, there were differing views about the timing of this session. Some parents preferred having the lived experience family at the start to foster connection and engagement. Others suggested having the lived experience family towards the middle or the end of the intervention, as the discussions within the group from earlier sessions could spark additional questions that could lead to deeper exploration and reflections. These preferences could have also been influenced by the timing at which the lived experience family was introduced, as in the first group, the lived experience family was introduced at the end, while in the second iteration it was introduced at the start.

*It was really amazing to have another family's perspective of their journey. That was really, really good. I really enjoyed that bit and it was good. It was at the start, so we have an idea what to expect. And kind of as a hope thing as well, I guess because they had a good result (Parent 3, Group 2)*

*I think it might work well there [final session] because we knew each other and we'd sort of discussed all of our various children, where we were with progress (Parent 2, Group 1)*

The importance of collaboration and bridging learning between parents and mental health professionals through the sharing of experiences and knowledge was also highlighted. Centring parents as experts in their child was important in empowering them to effectively care for their children.

*We are also the experts in our own kids, in their own behaviour, and whether people have to try and do it, manage it every day, and I think it was sort of quite refreshing to have a conversation rather than the kind of do this, and then having to come along and especially for [child’s name] coming onto sessions where it hadn't really worked and she hadn’t put on weight. (Parent 4, Group 1)*

#### Theme 2: From knowledge to practice

This theme encompasses parents’ reflections on how the group may have influenced their understanding of autism, AN, and their overlap. Again, insights gained from parental lived experience of caring for their children were highlighted. Enhanced understanding of autism and AN led to insights into broader family needs and relationships.

*Subtheme 2.1: Bridging the gap: Insights into autism and AN*

There were varying responses regarding the extent to which the group enhanced parents’ understanding of autism and AN. Several parents reported that discussions within the group enriched their understanding of autism and AN, while also acknowledging that this area is still in its infancy. Specifically, sessions with the dietitian were described by some parents as helpful in developing a better understanding of the impact of nutrition on the body and building flexibility around volume and variety in their young person’s intake, in addition to skills around eating socially in new environments. Others noted gaining deeper insights into the factors that have likely enhanced their children’s vulnerability to developing eating difficulties, such as differences in interoception and sensory processing. For some, being able to label these factors helped them to articulate and mentalise their children’s difficulties. This new learning led to reductions in parental anxiety and feelings of guilt and subsequently shifted parents’ approach in caring for their children.

*Maybe my child recovered from the anorexia, but anorexia was not the illness. It's not the root. The root is the trauma and the anxiety. (Parent 1, Group 1)*

*Some of the ways that we're trying to manage anorexia before it was not very helpful because of her autism, so I think I was insisting on having more variety and things like that for food and not being stuck on specific things, but obviously that's part of the autism. So, I should have, like, concentrate on different things than trying to worry about this type of thing basically, something like that as an example. (Parent 3, Group 2)*

Many parents found the content of the group to be of high quality, useful, comprehensive and well-structured, while concurrently, recognising that the facilitators were collaborative and flexible in their approach, as there were opportunities for discussion when relevant topics would arise. Perceiving the content of the group in a positive way likely motivated parents to engage more with the information, while the collaborative approach allowed them to gain insights that were more personally relevant to them.

*I think it was of a really high standard, as I said, it felt much more kind of high level, much more - like you’re involved in a discussion and presented with certain tools and certain ideas in certain ways of thinking about things that you can go away and experiment with and try out and just rather than kind of – and it did feel a bit more like, you know, we don't really know all the answers here, but here's a few things for you to try, and I quite like that approach. (Parent 4, Group 1)*

Some parents also said that learning and understanding more about the diagnostic overlap between AN and autism raised more questions than answers, which could leave them feeling confused at times. A few parents spoke about how their child rarely verbalised body image concerns, despite persistent restrictive eating behaviours. This left one parent feeling unsure about what was driving their child’s eating difficulties and, therefore, how best to approach management and support. One parent questioned whether all of their child’s eating difficulties would be better formulated within the context of autism only.

*She was diagnosed with anorexia, which I don't think ever was the right label for her and I think because she's autistic, I think it created problems for her, made her eating habits worse… (Parent 2, Group 1)*

There were also suggestions to further tailor the group to provide more personalised support. For example, there were varying preferences regarding the focus of the group, and whether this should be targeted towards autism, eating difficulties or a mix of both. There was also the suggestion to sub-divide the group based on children’s age, given that each developmental stage could present with different challenges around peer relationships, academic demands and independence.

*A few of the families came purely from the eating disorder point of view, whereas I think we were a bit more of the combination. (Parent 6, Group 2)*

*Subtheme 2.2: Putting learning into practice*

Improved understanding of autism and AN helped parents to adjust their approach at mealtimes. Parents described several practical strategies to support their children with their eating. Parents valued discussions around managing sensory sensitivities when choosing and mixing foods. Parents also spoke about the need to shift their own expectations around how much variety and flexibility their child may have in their food choices, whilst also balancing nutritional needs. Each parent described taking into consideration their child’s needs and tailoring their approach to facilitate eating.

*… from the group it was really trying to make it as easy as possible for her, sort of the neurodiverse mind, to be able to understand that she has to fuel herself, but sometimes understanding as well that maybe she just wants to eat one thing for dinner, and she'll eat a huge amount of it. (Parent 2, Group 1)*

*There seems to be like she’ll eat a lot of one kind of food, but doesn't really like mixing different food textures, flavours on the same plate. So, if you're trying to get a balanced diet, you know, normally you think about plate of food and about, you know, a certain amount of protein, a certain amount of carb, a certain amount of vegetables and salads or whatever. (Parent 4, Group 1)*

In some cases, the use of homework facilitated the recognition of their child’s needs and the implementation of strategies at home. However, there was a lack of confidence in using some of these tools, possibly due to insufficient guidance. Parents also described difficulties completing the homework and involving their child in this process due to busy family schedules and family dynamics.

*We were given some information and it's very difficult to - I don't, I'm not quite sure what to do now with the information and how to help and we were given some tools, but I'm not sure I feel confident still to manage on my own basically. (Parent 3, Group 2)*

*Oh, we weren't so good at doing the homework. I think it was just like life is busy and we didn't manage. (Parent 2, Group 1)*

Some parents said that having physical handouts (rather than electronic) made it difficult to retain all details, and in turn, to effectively share and/or implement learning at home. Consequently, parents suggested the possibility of sharing electronic booklets/handouts or a summary of what was discussed following each session to then revisit, as well as offering follow-up sessions to review progress and consolidate learning.

*I think it would have been quite nice to almost be given like a booklet of what we're going to work through each week. (Parent 6, Group 2)*

*I think there may be something about continuity, or supervision or record the people to see in which point they are, will be quite good and could be quite enriching for everyone. (Parent 1, Group 1)*

*Subtheme 2.3: Attending to broader family needs and navigating relationships*

Several parents reported that the content of the group helped them to reflect on their own strengths and differences, such as difficulties in identifying and articulating emotions, and to recognise and understand their own neurodiversity. This increased self-awareness, subsequently led to greater understanding, mentalisation and attunement, prompting parents to recognise and respond to their children’s emotional needs.

*The other thing, which I think is important, is to realise as well that apples don’t fall far away from trees. So, if all children are autistic, very likely someone else is autistic in this household. It could be me. (Parent 1, Group 1)*

For some, the group positively influenced relationships by fostering better communication and collaboration among family members, ultimately enhancing the overall family dynamics.

*I think one of the big things that's come out of it is that me and him [partner] are communicating much better about things. And you know, if either of us is having a bad day, we're finding something particularly hard or, you know, [child’s name] isn't really challenging. We're talking to each other much more about it and I think. We spoke before, we've been close, but I don't think we would have spoken as we do now. So, I think that's been a real positive of communication between us as well as been really, yeah, that's been a real positive. (Parent 6, Group 2)*

A few parents were interested in having the option for their children to either form a group or join their sessions, as they sought to avoid creating a sense of separation or secrecy between themselves and their children. However, there was also uncertainty about whether this would be feasible, as some children may not be interested, or it might not be appropriate for them to join due to the sensitive nature of some discussions.

## Discussion

### Main findings

The current study aimed to provide an overview of a group intervention for parents of young people with AN and suspected or diagnosed autism. The primary aim was to examine its feasibility and acceptability, and to understand parents’ experience of attending the group to inform future intervention development.

Data suggests the intervention was feasible and acceptable. Most families attended consistently. When both parents struggled to attend due to work and caring demands, usually one was able to attend and share the learnings with the other parent(s), suggesting positive engagement. High scores were observed on session-by-session ratings of group relevance, usefulness and improvements in parental understanding of their child.

Qualitative findings showed that the group improved some parents’ understanding of autism and AN and provided them with practical skills to manage at home. Often, new knowledge came through honouring parental expertise and learning from one another. Parents also described a sense of relief and gratitude in being able to connect with other parents and mental health professionals. This was described as leading to reductions in self-blame and isolation and empowering parents to effectively care for their children. This is in line with previous research demonstrating that group interventions can improve parents’ understanding and confidence to manage their child’s eating difficulties and that introducing these groups early in their journey can improve outcomes [2, 8, 9, 27, 52, 55, 60, 61]. While generally described as helpful, some parents noted that the intervention was not tailored enough to their specific needs, either because they were further along their journey, or the focus was not on the specific aspects they sought. This may speak to the importance of facilitators setting clear expectations around the aims and scope of the group from the outset with families. In contrast, parents reported that previous experiences of standard care were not always sufficient for their child, and that the group was a valuable addition in ensuring that they received appropriate and tailored support. This aligned with previous research demonstrating that current mental health services may not always be fully meeting the needs of autistic people and their families, often due to a lack of knowledge, resources and skills in identifying and supporting autistic people and the lack of clear guidance on when and how to adapt treatments [2, 6, 35–37].

Parents also described several barriers to attending group, including busy schedules, time constraints and other caring demands. Parents provided several key recommendations for future developments of the intervention, including offering the group at an earlier time point in treatment, introducing follow-up sessions, developing and sharing materials from the beginning, and offering remote sessions for those who are unable to attend in person. This is consistent with findings from a recent systematic review that identified the use of handouts, perceived parental confidence with the intervention content and feeling supported/accepted by the group to be facilitators to acceptability [56]. There was also discussion of whether online or face-to-face format would be more appropriate. Previous data suggests parents, more so than young people, prefer online eating disorder care, however people do question whether treatment is as effective or engaging when delivered online [10, 17, 63]. Furthermore, the use of homework appeared to be helpful in supporting parents apply the learning at home. However, there were reported difficulties completing homework exercises due to family pressures and time constraints. Previous research has demonstrated that the use of homework can improve engagement, with greater homework compliance leading to better outcomes [32, 44, 48]. Parents of autistic CYP with AN have also reflected on positive the benefits of targeted resources as part of an intervention [3]. Given the high caregiving demands of this population, our findings highlight the need for flexibility and realistic expectations around the use of homework, while still supporting with skill acquisition and generalisation beyond the group intervention.

The group led some parents to question whether their child met diagnostic criteria for AN or whether there was a more applicable label to explain their eating difficulties. Previous research has shown that autistic women with a diagnosis of AN reported a lack of concern about body image, suggesting that AN may present differently in autistic girls and that sensory differences may be more involved in the development and maintenance of eating difficulties [6, 15]. Furthermore, eating difficulties are a common part of autistic presentations, and do not necessarily indicate co-occurring AN [7], hence it is key to ensure the group is being offered to the right cohort. Considering that autistic CYP with AN exhibit distinct characteristics compared to those with AN alone, it is essential to develop a better understanding of phenotypic and diagnosis profiles to facilitate accurate diagnosis and improve care pathways. For our intervention, it is important to reflect on how facilitators conveyed this complex understanding to parents and to consider the extent to which this helped to contain, rather than amplify, parental anxiety. However, perhaps it is more so the priority that (regardless of diagnosis) parents have the knowledge and tools to adapt their response to their child’s identified traits and needs.

### Clinical implications and future directions

Findings suggest that there is a clear need for a group intervention to support parents of autistic young people with AN. Parents have reflected upon the potential benefits of offering the intervention earlier. It is possible that this could reduce parental anxiety, perceived isolation and self-blame early in treatment, allowing for parents to be better supported and care more effectively for their child. However, given the intensity inherent in phase 1–2 of FT-AN treatment, with parents tasked with supporting around managing meals, weight restoration, and generally supporting their young person to safely manage distress, it may not be possible for parents to prioritise a group like this one. However, this does speak to the importance of ensuring FT-AN treatment is adapted to meet neurodiverse needs, particularly as many parents reflected the standard treatment is not effective for their family. Adapted FT-AN treatment for autistic young people has been described elsewhere (c.f., [47]) and is beginning to be implemented. Future research should evaluate the acceptability, feasibility and effectiveness of outcomes with these treatment adaptations.

This is an initial evaluation with promising findings that requires further testing. A controlled study design with a larger sample is now needed. Future research should also investigate the effects of the group intervention on parent’s levels of distress, expressed emotion, caregiving burden and sense of competency, as these have been found to influence treatment response and outcomes [12, 18, 26, 58].

### Strengths and limitations

As far as we are aware, this is the first study to evaluate the acceptability, feasibility and pre-post changes of a group intervention for parents of autistic young people with AN in a specialist child and adolescent eating disorders service. A key strength relates to the fact that many families had both parents attending sessions.

However, this study has several limitations. First, the sample size was small, making the generalisability of the findings limited. Second, given that only a subgroup agreed to participate in qualitative interviews, it could be that these parents either had better experiences and thus were more inclined to share these with the team or worse experiences and therefore were more motivated to share their thoughts. Third, most participants were White British with sufficient resources to take time off work to attend the group. As a result, we cannot determine whether the group is acceptable to families from more diverse backgrounds.

## Conclusion

This group intervention for parents of autistic young people with AN appears to be acceptable and feasible. It can also help parents feel more supported and empowered and provide them with skills to effectively care for their children. Further research is needed to investigate the potential effectiveness of the group intervention. Recommendations for future autism and eating disorder parent groups include offering this group at an earlier timepoint in treatment, introducing follow-up sessions and developing and booklets that contain information and practical skills to manage at home.

## Supplementary Information

Supplementary Material 1.

## Data Availability

The datasets used and/or analysed during the current study are available from the corresponding author on reasonable request.
