# Supplementary material for: A pilot study investigating the acceptability and feasibility of a group intervention for parents of autistic young people with anorexia nervosa within a specialist eating disorders service
Source: J Eat Disord. 2025 Sep 29;13:217. doi: 10.1186/s40337-025-01399-4 (PMC12482359; doi:10.1186/s40337-025-01399-4)
Supplement: Supplementary file 1 — Supplementary Material 1. [file 40337_2025_1399_MOESM1_ESM.docx]

**Qualitative interview**

**Expectations**

- What motivated you to participate?
- What were your expectations or hopes before attending the parent group?

**Experience of the group**

- Can you describe your overall experience with the group? (prompts: what was helpful, what was unhelpful, what have you taken away from this experience, what insights, if any, did you gain from participating?)
- How did the group meet or differ from your expectations?/How did the group compare to what you had in mind?
- What are your reflections on the information provided in the group?
- What are your reflections on the content taught in the group?
- What are your reflections on interacting with the other parents / staff?

**Challenges and difficulties**

- Were there any aspects of the group that you found challenging or difficult to grasp?

**Areas to improve on**

- Were there any additional resources or support you would have liked to see in the group?
- Was there any topic or issue that you believe should have been discussed in the group but wasn't? If so, what was it?
- What suggestions do you have for improving the group?
